# Factors Influencing Residents’ Behavior in Internet Recycling: From the Perspective of the Adoption of New Technology

**DOI:** 10.3390/ijerph19106166

**Published:** 2022-05-19

**Authors:** Tingting Liu, Zichen Zheng, Zhichao Wen, Shangyun Wu, Yaru Liu, Jing Cao, Zhixiong Weng

**Affiliations:** 1 Institute of Circular Economy, Beijing University of Technology, Beijing 100124, China; tingting.liu@bjut.edu.cn (T.L.); dingding@emails.bjut.edu.cn (Z.Z.); wushangyun@emails.bjut.edu.cn (S.W.); caojing@emails.bjut.edu.cn (J.C.); 2 State Information Centre, Beijing 100045, China; wenzc@sic.gov.cn; 3 Price Waterhouse Coopers, Beijing 100020, China; yaruliu100124@163.com

**Keywords:** internet recycling, recyclable resource, UTAUT, behavioral intention, recycling behavior

## Abstract

Achieving carbon neutrality has become a major national strategy for sustainability, and the recycling of recyclable resources is an important direction toward doing so. Due to the huge amounts of recyclable resources generated every year and the low recycling rate, a new Internet recycling model with great potential to increase the recycling rate has developed rapidly in China. However, low participation from residents hinders the sustainable development of Internet recycling. Through this study, we aim to uncover potential avenues for improving Internet recycling behavior. The factors influencing Internet recycling from the perspective of new technologies have scarcely been investigated. Therefore, this study used the Unified Theory of Acceptance and Use of Technology theoretical framework to explore the factors influencing residents’ intentions and behavior toward Internet recycling. A questionnaire survey was conducted with 500 residents of Beijing, China, and empirical analysis was conducted using the structural equation model. The results indicated that social influence and performance expectancy significantly influence residents’ intentions to participate in Internet recycling, whereas effort expectancy and perceived risk do not. Facilitating conditions and behavioral intentions were identified as influential factors for use behavior. Relevant recommendations for promoting residents’ Internet recycling behavior were proposed.

## 1. Introduction

The global response to climate change is unprecedentedly urgent today. Carbon neutrality is an important concept in tackling climate change and achieving sustainable development. To address climate change and reduce total greenhouse gas emissions—mainly carbon dioxide (CO_2_)—37 countries, including China, have formally committed to carbon neutrality through national laws, agreements, or policy declarations, and another 52 countries have made verbal commitments [1]. China has pledged to achieve carbon peak (the country’s highest carbon emissions point, followed by decreasing emissions) by 2030, and carbon neutrality by 2060. Recycling of recyclable resources is an important step toward achieving carbon neutrality, as recyclable resources can help to reduce carbon emissions [2]. For instance, recycled paper can save 60–70% of energy consumption, and reduce air pollution by 60–70%. Its greenhouse gas emission reduction efficiency is 5.42tCO_2_e per ton of waste paper. Recycled copper can save 87% of energy consumption, and its greenhouse gas emission reduction efficiency is 14tCO_2_e per ton of waste copper [3].

With consumption increasing and the national economy continuing to develop, huge amounts of recyclable resources are generated every year, and present a rapid growth trend in China. According to the “Recyclable Resource Recycling Industry Development Report of China”, as of 2020, the total amount of recyclable resources in 10 major categories recycled in China reached 380 million tons, representing an increase of 7.3% from 2019 [4]. Recyclable resources generated in the high-consumption megacity Beijing increase at an average annual rate of 8% [5]. In addition, with the rapid economic growth, natural resources are consumed in large quantities, and sustainable development of the economy is constrained by these resources. Thus, developing resource recycling practices can effectively alleviate the shortage of resources in China. Furthermore, it would reduce environmental pollution and carbon emissions, and promote “green” development. Therefore, resource recycling is crucial.

Some developed countries have formed relatively complete recycling systems for household recyclable resources through legislation, and their relevant policies and methods reflect advanced resource management ideas. The United States has adopted a model that combines government policy support and active public participation in the development of the recycling industry. The U.S. enacted the Resource Conservation and Recovery Act in 1976, and more than half of its states have enacted some form of recycling regulations [6]. Germany was one of the first countries to develop a circular economy, and has since formed a relatively complete legal system to support it, including laws, regulations, and guidelines. To support implementation of these regulations, non-governmental organizations for resource recycling—such as the Duales System Deutschland recycling system—have gradually come to be formed [7]. Japan has a strict and elaborate process for waste separation and recycling through a legal system that binds the responsibilities and obligations of localities, companies, and citizens in the recycling chain. The waste collection rate in Japan is almost 100%, achieving a high level of public participation [8]. By contrast, the development of recycling in China has considerable room for improvement compared with developed countries.

Collection is the key to recycling. However, constructing collection systems has been the weakest link in the comprehensive utilization of recyclable resources in China [9]. The recycling rate of China’s recyclable resources was less than 60% in 2018 [10]. In China, the traditional recycling industry relies mainly on scavengers and curbside recyclers, who constitute more than 90% of the industry’s workforce [11]. This forms a large-scale, informal, reverse-logistics system that is fragmented and has an insufficient scale of effect and low resource utilization rate, which can cause serious secondary pollution [12]. Moreover, with the advent of the digital era and changes in residents’ consumption habits and daily routines, traditional recycling is no longer well adapted to the development goals of large consumer cities, such as the construction of ecological civilizations and carbon neutrality goals.

In this context, Internet technology and the Internet of Things have been widely introduced into the field of resource recycling. Many companies have begun to focus on developing Internet recycling to improve the recycling capacity and efficiency of recyclable resources, forming a new Internet recycling model. The Internet recycling model is a revolution of traditional recycling, which integrates the concept, technology, and mode of the Internet into the recycling process [13]. Internet recycling connects online and offline services by building Internet recycling platforms, and has several advantages over traditional recycling. First, Internet recycling can effectively reduce the information asymmetry between buyers and sellers to achieve information disclosure. Second, residents can use Internet recycling platforms to arrange for door-to-door recycling, which is faster and more convenient. Finally, Internet recycling is more standardized, as recyclers are trained to provide professional services to residents, and the platform tracks recycled resources to ensure that they flow into formal disposal channels [9].

In recent years, the Chinese government has strongly supported the development of Internet recycling. On 14 April 2015, the National Development and Reform Commission of China issued the “2015 Circular Economy Promotion Plan”, proposing that China should promote and guide the innovation of recycling models for recyclable resources, and emphasizing the need to explore the Internet recycling model. Subsequently, the “State Council’s Guiding Opinions on Actively Promoting the Internet Plus Action” and the “Internet Plus Three-Year Implementation Plan for Green Ecology” have been issued, which encourage Internet companies to actively participate in urban waste recycling, platform construction, and innovation of the recycling model. On 10 May 2016, six ministries of China, including the Ministry of Commerce and the National Development and Reform Commission, jointly issued the “Opinions on Promoting the Transformation and Upgrading of the Recyclable Resource Recycling Industry”, emphasizing that recycling companies should establish a new concept of Internet Plus development, which advocates for industries to use the platform of the Internet as a basis to achieve interconnection, create new business models, and promote industry transformation, thereby bringing innovation to the recycling model and its organization. This encourages Internet recycling companies to extend their business through mobile phone applications—such as WeChat—and websites to achieve the organic combination of online and offline recycling. On 31 January 2019, Shanghai promulgated the “Regulations of Shanghai Municipality on Waste Management” to implement mandatory garbage sorting; this document recommends the use of recycling methods such as Internet Plus recycling. On 7 July 2021, the National Development and Reform Commission issued the “14th Five-Year Plan” for the development of China’s circular economy, noting that the Internet Plus recycling model should be actively implemented to achieve online and offline synergy and enhance the integration ability of standardized recycling companies with informal operators, improving convenience for residents in recycling resources. While the government actively promotes the integration of the Internet Plus and recycling industries, some recycling companies have already adopted the Internet recycling model. For instance, China’s lead recycling company, Aibolu, built an Internet platform to efficiently interact with sellers, manufacturers, and disposers in the flow of information and materials, expanding from a recycler to a comprehensive service provider in the recycling system. Beijing Sanitation Group utilizes Internet technology to explore a smart garbage-sorting model that integrates garbage removal and resource recycling networks, deploying intelligent recycling cabinets to communities that, in turn, collect data and establish an integrated service network platform to provide real-time rewarding feedback regarding residents’ independent delivery behavior.

Although Internet Plus recycling projects have obtained some achievements, several problems have occurred in their development process. For instance, some residents have disputes regarding the valuation of recyclable resources and concerns about information security [14]. Most importantly, residents do not have a sufficient understanding of Internet recycling, leading to low participation and lack of enthusiasm [10,12,15], which has caused many online recycling platforms to face the risk of bankruptcy and encounter other operational difficulties [16,17]. Therefore, while the government and companies are actively promoting the Internet recycling model, there is an urgent need to explore the status quo and the factors influencing residents’ participation in Internet recycling of recyclable resources. Identifying the key influencing factors and paths would reveal effective measures to promote Internet recycling and maintain the healthy operation of the industry. Internet recycling is a new model for the Internet era, and previous studies have begun to focus on these factors. Wang et al. [18] developed an extended theoretical framework of planned behavior to explore residents’ willingness to participate in online electronic waste recycling, along with its influencing factors. The results demonstrated that subjective norms, attitudes, perceived behavioral control, and economic motivation had significant positive effects on intentions to participate in Internet recycling. Some studies combined the theory of planned behavior (TPB) with other theories, such as the technology acceptance model (TAM) and perceived risk (PR), to construct an integration model of factors influencing residents’ participation in Internet recycling. These studies revealed positive effects of attitude, subjective norms, perceived usefulness, and perceived benefits, although PR had a negative effect on the intentions of millennials toward the adoption of the platform [10,19,20]. Existing studies on factors influencing residents’ recycling behavior are mostly based on the TPB or TAM. However, few studies have examined the use of Internet recycling platforms. Therefore, this study uses the unified theory of acceptance and use of technology (UTAUT) model to innovatively explore the factors influencing residents’ participation in Internet recycling from the perspective of the adoption of new technology. The UTAUT is an integrated model proposed through the combination of eight theories of individual behavior (the theory of reasoned action (TRA), TAM, motivational model (MM), TPB, combined TAM–TPB (C-TAM-TPB), model of PC utilization (MPCU), innovation diffusion theory (IDT), and social cognitive theory (SCT)) and empirical verification. It explains up to 71% of usage behavior [21], which is higher than previous models [22]. Moreover, the UTAUT model has been widely used to study the factors influencing the adoption of new Internet technologies, such as online shopping [23,24,25], online exam systems [26], mobile payments [27,28], and mobile banking [29]. Therefore, this study used the UTAUT model to analyze the factors and obstacles that affect residents’ participation in Internet recycling. This study focuses on Beijing—a representative city in China—as it is a high-consumption megacity with a high level of Internet coverage and development compared to remote and rural areas. This study focuses on how to promote Internet recycling participation among residents in such cities, proposes policy recommendations, and provides a reference for promoting the development of Internet recycling and contributing to the achievement of China’s carbon neutrality goals.

## 2. Theoretical Basis and Research Hypotheses

**Figure 1:**
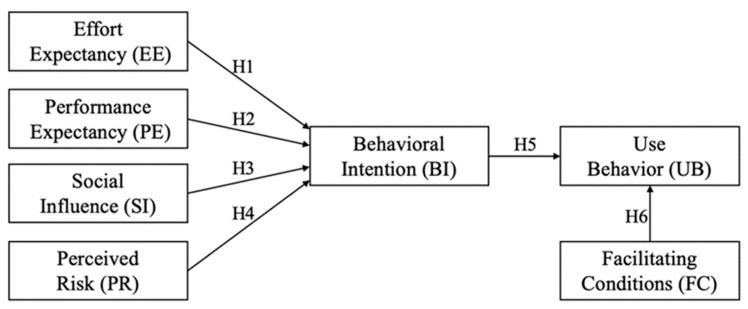
Theoretical framework.

In this study, we sought to increase the resident participation rate in Internet recycling. The UTAUT model, developed by Venkatesh et al. [22], was adopted as the theoretical framework. The UTAUT model involves a wide range of disciplines, including sociology, psychology, and the behavioral approach to information systems. It is an integrated, causal model developed for describing and predicting the acceptance and use of technology in various fields, and has been empirically tested for validity and reliability [30,31]. The UTAUT integrates eight models that identify factors determining individuals’ acceptance of technology. It contains six variables, with effort expectancy (EE), performance expectancy (PE), and social influence (SI) proposed as direct determinants of individuals’ behavioral intention (BI), and facilitating conditions (FC) and BI identified as key predictors of individuals’ use behavior (UB) [22,32]. This study used these variables as well as PR, which is derived from the PR theory developed by Bauer [33], to explain consumer behavior. PR is a common extension of the UTAUT, as many new products, including Internet recycling platforms, are considered inherently risky [27]. The research model is shown in Figure 1.

### 2.1. Effort Expectancy

EE is defined by Venkatesh et al. [22] as the degree of ease in adopting a technology. The use of a new online technology can be challenging, but the application interface design for online recycling is actively working toward improved ease of use. As such, EE may be one of the key factors of BI toward using the technology. EE is generally directly proportional to residents’ BI [34]. In this study, EE refers to the degree of ease associated with residents’ adoption of the Internet recycling platform. The more difficult it is to use the Internet recycling platform, the lower the residents’ acceptance of Internet recycling. Therefore, the following hypothesis was proposed:

**Hypothesis** **1** **(H1).** *EE has a negative effect on residents’ BI to participate in Internet recycling.*

### 2.2. Performance Expectancy

PE refers to the perceived usefulness of adopting a technology, and the belief that the use of the adopted technology will be helpful in one’s job performance [22]. PE is generally directly proportional to residents’ BI [35]. It has been shown that PE positively affects technologies that provide residents with quicker and more efficient services [27]. In this study, PE refers to the usefulness of the Internet recycling platform, as it can provide services to meet residents’ needs and convenience to participate in resource recycling. Therefore, the following hypothesis was proposed:

**Hypothesis** **2** **(H2).** *PE has a positive effect on residents’ BI to participate in Internet recycling.*

### 2.3. Social Influence

SI refers to the degree to which an individual feels influenced by surrounding groups [22]. A study found that SI is directly proportional to residents’ BI [36]. In this study, SI refers to the degree of influence of those that residents consider important—such as families and friends—on their adoption of Internet recycling. As residents are increasingly influenced by surrounding groups, their intention to participate in Internet recycling increases. Therefore, the following hypothesis was proposed:

**Hypothesis** **3** **(H3).** *SI has a positive effect on residents’ BI to participate in Internet recycling.*

### 2.4. Perceived Risk

Tarpey and Paul [37] classified PR into financial and time risks. Korgaonkar and Wolin [38] emphasized that security and privacy are important factors of PR in the Internet environment. When the PR is high, residents may not have a positive recycling attitude [39]. In this study, PR mainly refers to privacy, financial, and time risks that the Internet recycling platform may cause. With the increase in PR, residents’ intention of using Internet recycling decreases. Therefore, the following hypothesis was proposed:

**Hypothesis** **4** **(H4).** *PR has a negative effect on residents’ BI to participate in Internet recycling.*

### 2.5. Behavioral Intention

In this paper, BI refers to the residents’ intent to use Internet recycling in the future under certain conditions. UB refers to the actual adoption of Internet recycling by residents. Therefore, the following hypothesis was proposed:

**Hypothesis** **5** **(H5).** *BI has a positive effect on residents’ UB to participate in Internet recycling.*

### 2.6. Facilitating Conditions

FC refers to “the degree to which an individual believes that an organizational and technical infrastructure exists to support the use of the system” [22]. In this study, FC refers to residents’ perceptions of various organizational and technical support conditions required for the smooth use of Internet recycling. Theoretically, the impact of FC on residents’ UB toward adopting online platforms is supported by previous studies [26,29]. Therefore, the following hypothesis was proposed:

**Hypothesis** **6** **(H6).** *FC has a positive effect on residents’ UB to participate in Internet recycling.*

## 3. Materials and Methods

Beijing is the capital of China, and a megacity with a resident population of over 21 million. Promoting the participation of Beijing residents in Internet resource recycling has been a key concern of the Beijing Municipal Government. Therefore, this study chose Beijing as the case study area, and conducted a questionnaire survey on its citizens regarding their usage behavior of Internet recycling.

### 3.1. Questionnaire Design

The questionnaire was designed based on the proposed hypotheses and expert consultations. It consisted of two parts: (1) residents’ demographic information, including gender, age, and education; and (2) residents’ usage behavior of Internet recycling. The questionnaire included 23 questions (Table 1) to measure 6 variables, which were constructed based on the UTAUT, with some factors and wording modified to better reflect the uniqueness of Internet recycling. Answers were rated on a five-point Likert scale (1 = strongly disagree, 5 = strongly agree).

### 3.2. Sample and Data Collection

Before the formal distribution of the questionnaires, 100 pilot questionnaires were administered to the residents in Beijing via the Internet, to test the rationality of the questionnaire design. After obtaining the pilot questionnaire data, some questions that seemed misleading or ambiguous were revised to make them more in line with the purpose of the research. The formal questionnaires were distributed randomly by stratified sampling based on the proportion of the resident population in each of the 16 districts and counties in Beijing. The distribution of the questionnaire sample is shown in Table 2. A web questionnaire platform was used to distribute the questionnaires. The quality of the questionnaires was ensured by limiting the minimum response time, ensuring that each questionnaire was individually coded and traceable, and ensuring the completeness of the responses. In case of invalid questionnaires, additional questionnaires were distributed. A total of 500 valid questionnaires was collected. The survey was ethically conducted, and all respondents participated voluntarily.

### 3.3. Data Analysis

This analysis employed structural equation modelling (SEM) to examine the hypothesized relationships between the constructs. First, the reliability of the questionnaire was analyzed. Then, confirmatory factor analysis (CFA) was conducted, and the measurement model was evaluated along with convergent validity, composite reliability, and content validity. Subsequently, the proposed hypotheses were assessed. Statistical analyses using the SEM were conducted with SPSS (v. 22) (International Business Machines Corporation, New York, NY, USA), as well as its supporting modeling software, AMOS (v. 22) (International Business Machines Corporation, New York, NY, USA).

## 4. Results

Based on the methods and data introduced above, we conducted our empirical analysis. The results are presented below.

### 4.1. Descriptive Statistics

The main features of the respondents (gender, age, and education) are described in Table 3. Overall, 240 (48%) of the respondents were men and 260 (52%) were women. Most were aged 19–34 years (n = 170, 34%) and 35–49 years (n = 145, 29%), indicating that the age distribution of the survey samples was reasonable.

### 4.2. Measurement Model Analysis

#### 4.2.1. Reliability Analysis

Reliability was tested using Cronbach’s alpha to determine the internal consistency and reliability of the constructs. According to Nunnally [40] and Cronbach [41], a questionnaire has good reliability when Cronbach’s alpha is above 0.7. Table 4 shows that the Cronbach’s alpha for all constructs was above 0.7, demonstrating that the data were reliable and the reliability of the scale was good. Furthermore, all indicator loadings were above 0.5 [42], indicating internal consistency.

#### 4.2.2. Validity Analysis

A validity test reflects the degree of effectiveness of the survey data. The better the measure matches the content to be examined, the higher the validity. First, the test of Bartlett’s sphericity and the Kaiser–Meyer–Olkin (KMO) test were used. The *p*-value of Bartlett’s sphericity test was zero, denoting that it was significant. The value of KMO was 0.892, which was greater than the critical value (0.7). This indicated that the original indicators were suitable for further factor analysis. A CFA using SPSS 22.0 (International Business Machines Corporation, New York, NY, USA) was used to estimate the measurement model. Convergent validity was used by average variance extracted (AVE). As shown in Table 4, AVE values ranged from 0.511 to 0.620, and were above the threshold value of 0.50 [43], indicating that the convergent validity of the measurement model was satisfactory. In addition, CR values exceeded the suggested value of 0.70 [43], except for BI. The latter remained greater than 0.6 (a proposed threshold value) [44], indicating composite reliability. Moreover, a review of previous research and consultation with experts in the field strengthened the content validity. The measurement model had satisfactory validity.

### 4.3. Structural Model and Hypothesis Testing

The overall model fit was tested. Commonly used indices with proposed reference values and fit results are shown in Table 5. All fit indices indicated a good overall model fit. Moreover, the value of CMIN/DF was 1.891, demonstrating that the model had a fine adaptation to the actual sample data.

**Figure 2:**
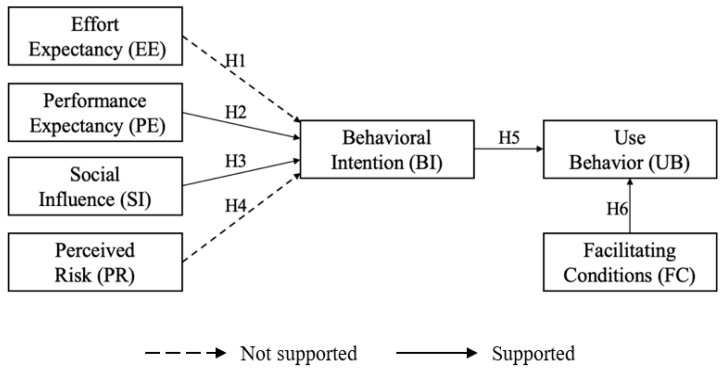
The simulation path diagram of the structural equation model.

Next, the research hypotheses were tested. The results indicated that the effects of SI and performance expectation on BI, and the effects of BI and FC on UB, were significant. However, the relationships between EE and BI, and between PR and BI, were not significant. The results of the model are shown in Table 6 and Figure 2.

## 5. Discussion

The results supported H2, H3, H5, and H6, but not H1 or H4.

The statistical results provide strong proof of the causal path between PE and BI, indicating that Internet recycling platforms have greater relative advantages over traditional recycling methods in terms of saving time, improving efficiency, providing valuable information, and providing targeted services. Previous studies have revealed specific comparative advantages of Internet recycling platforms relating to PE. E-waste collection is more advantageous in its scope of collection, convenience of service, accessibility, and flexibility, providing higher PE for residents [52,53,54].

The empirical results supported a significant relationship between SI and BI toward Internet recycling. As Internet recycling is an emerging technology, residents tend to be cautious toward it, and their BI is often influenced by important people around them. However, a previous survey revealed that although 97.8% of residents in Beijing gave satisfactory feedback after using the Internet platform for recycling, 82.4% of residents said that they did not actively recommend the Internet platform to their family, friends, or colleagues [55], which may be one reason for the low participation rate in Internet recycling. In addition, Internet recycling users are mostly young and middle-aged people with a high level of education, and they are more receptive to environmental protection concepts such as recycling and garbage sorting. These characteristics make it easy for SI to have a greater impact on residents’ BI toward Internet recycling, which is a common phenomenon for a series of other Internet Plus products, such as Internet Plus mobile payment [56] and Internet Plus finance [57].

As expected, FC was a crucial factor influencing residents’ participation in Internet recycling. Internet recycling is more popular among young people, most of whom are progressing in their careers, and are more sensitive to time compared to other age groups. Therefore, the convenience of Internet recycling platforms directly influences its adoption by residents, compared to other factors. The importance placed on FC has been frequently mentioned as an influential factor in recycling [58,59,60,61].

As proposed in the conceptual model, statistical results indicated a significant path between residents’ intention to participate in Internet recycling and their actual behavior. A previous survey revealed that most residents expressed interest in using the Internet recycling platform, and were willing to actively search and learn how to use it [55], indicating that positive BI positively influenced residents’ participation in Internet recycling behavior. Therefore, this suggests a good foundation for increasing the participation rate of Internet recycling among Beijing residents.

EE did not account for any statistical variance in the BI to participate in Internet recycling. Venkatesh et al. [22] suggested that EE is stronger among users at early stages of the experience, suggesting that the impact of EE on residents decreases with the use of Internet recycling platforms. Wang [62] also indicated that EE among residents with no experience in Internet shopping had a stronger influence on BI. Our findings were consistent with this inference. In addition, related companies continue to explore the simplicity of the Internet recycling platform system to lower the threshold of use for residents, which is another reason for EE not being a significant influencing factor. Therefore, H5 was not supported.

PR did not account for any statistical variance in BI to participate in Internet recycling, in contrast to previous studies. Although the development of online businesses has increased residents’ awareness of protecting personal privacy and ensuring transaction security, third-party mobile payment platforms such as WeChat and Alipay are more mature today than they were previously, and are supervised by sound laws and regulations; consequently, residents are less worried about the privacy and financial risks that can arise from online transactions. Furthermore, some online recycling platforms in China have a higher level of consumer trust than others, as they cooperate with large online shopping malls, such as the cooperation between All Things Renew and Jingdong Online Mall—China’s leading online recycling company and online shopping mall, respectively. Therefore, consumer trust in the online mall is transmitted to the recycling platform. This further reduces residents’ concerns regarding privacy and financial risks associated with online recycling transactions. Internet recycling platforms are distinguished from platforms such as financial product trading [63] and e-commerce [64,65], due to smaller transaction amounts and reduced amounts of shared personal information. Therefore, the impact of PR on residents’ intention to adopt Internet recycling platforms is smaller.

## 6. Implications for Practice

### 6.1. Leveraging Comparative Advantages to Meet PE and Exploring a Good Profit Model

The findings indicated that PE had a positive effect on residents’ willingness to participate in Internet recycling, and Internet recycling platforms had a comparative advantage in raising PE. Therefore, Internet recycling platforms should use their own advantages to provide residents with transparent recycling information, reduce the cost of information communication between buyers and sellers, and offer appropriate collection pricing that meets residents’ expectations to promote willingness to recycle [66]. In recent years, many domestic recycling companies have started to explore Internet recycling. However, these companies have strong homogeneity, and the profit space in the industry is small, requiring financing or government subsidies to maintain operations. Many newly established Internet recycling companies face the threat of bankruptcy [67]. Therefore, Internet recycling companies should make full use of modern network technologies—such as the Internet of Things, big data, and cloud computing—to collect and analyze user feedback. Companies can provide personalized platform services by collecting users’ age, preferences, and other information to form their own unique competitiveness. Internet recycling companies should explore good profit models and avoid over-reliance on government support.

### 6.2. Developing Heterogeneous Publicity Strategies to Expand the Impact of SI

Our findings demonstrated that SI had a positive impact on residents’ willingness to participate in online recycling, and that current SI requires urgent improvements. Internet recycling companies should make use of both online and offline channels for publicity, including using WeChat, QQ, and other online social tools, creating sharing links, and providing residents with sharing rewards to encourage their sharing behavior. Offline, enterprises should regularly organize employees to distribute leaflets and set up roll-up banners in crowded places. The combination of online and offline publicity promotes the exchange of information between existing users and potential users of the Internet recycling platform, thereby improving the effect of SI. In addition, most of the residents participating in Internet recycling are young and middle-aged, and their income and education levels are generally high. Most people participating in recycling at home are middle-aged or elderly. Therefore, to increase the participation rate of Internet recycling, companies should utilize heterogeneous publicity methods based on the characteristics of different user groups. For young and middle-aged users, online publicity should be used to retain existing users and produce youthful, personalized, and fashionable publicity content. To increase participation among middle-aged and elderly groups, Internet recycling companies should set up service personnel at offline recycling sites to help elderly groups solve technical problems, such as difficulty in using smartphones and smart recycling machines, to improve enthusiasm for their Internet platforms.

### 6.3. Strengthening the Construction of Offline Supporting Facilities and Integrating Informal Recyclers to Create FC for Internet Recycling

The findings revealed that FC had a positive impact on residents’ Internet recycling behavior. To improve FC, Internet recycling companies should strengthen the construction of offline supporting facilities, such as intelligent recycling machines, and provide professional and considerate services in the overall process from appointment to transaction, to increase user satisfaction and loyalty [66]. Moreover, Internet recycling companies can cooperate with local communities and properties to establish recycling sites. In improving the convenience of Internet recycling, stakeholders should consider the integration of informal recyclers. In China, although traditional informal recyclers do not consider the environmentally sound disposal of recycled products to maximize profit, they usually have extensive experience in recycling, and are familiar with the areas in which they work [68]. Thus, the integration of informal recyclers into the Internet recycling system can greatly improve recycling efficiency and provide added convenience for residents. Internet recycling companies could absorb traditional informal recyclers and conduct standardized training and unified management. For instance, Incom Recycle integrates traditional informal recyclers into its Internet recycling platform, providing them with timely and extensive recycling information, while allowing individual recyclers to remain responsible for their original offline area. Traditional informal recyclers do not require payment by the company, and use the Internet platform to increase their recycling business. In turn, the company expands the scope of their recycling area.

### 6.4. Increasing Financial Support and Optimizing the Policy Environment

In addition to the above recommendations, the appropriate development of Internet recycling cannot be achieved without the support of the government. At present, most of China’s Internet recycling companies are private, with short establishment time and small initial scale, often facing problems such as high operating costs, low profit levels, and a lack of good profit models; thus, their development cannot be achieved without government policy support and financial subsidies. Therefore, the government should help the Internet recycling industry by summarizing existing practices, analyzing successful cases, and supporting the innovation of profit models of Internet recycling platforms. In the short term, the government could provide tax incentives and financial subsidies for Internet recycling companies to stimulate enthusiasm. In the long term, it is necessary to formulate relevant policies and regulations to promote the transition from charging to paying for the recycling and treatment of recyclable resources, which is a necessary stage to promote the long-term sustainable development of the Internet recycling industry. Simultaneously, government departments should strengthen residents’ education on environmental protection through the Internet, television, newspapers, bulletin boards, and other media to cultivate residents’ awareness of sorting and recycling of household waste. Although China has formulated many development plans and guidelines to promote the healthy development of the recycling industry in China, the implementation details of laws and regulations on the development of the Internet recycling industry have not yet been written. Therefore, the government should take overall control of recyclable resource management systems, promote the combination of traditional and Internet recycling, improve the revision of relevant technical standards for recycling, sorting, transportation, and processing, and implement unified guidance and management. Moreover, it is necessary to crack down on illegal recycling sites to standardize the development of recyclable resource management systems and promote the long-term progress of the Internet recycling industry.

## 7. Conclusions

This study examined the factors influencing residents’ participation in Internet resource recycling in Beijing. This study was based on the UTAUT, and added PR as an independent variable to construct a model of factors influencing residents’ participation in Internet recycling. Subsequently, a questionnaire was distributed to residents in 16 districts and counties in Beijing to investigate the current situation of Internet recycling and obtain model data. Finally, a structural equation model was built to analyze and process the questionnaire data. The results demonstrated that SI and PE positively affected residents’ intention to participate in Internet recycling, while FC and BI positively affected residents’ behavior of participating in Internet recycling, whereas EE and PR did not have significant effects on residents’ intention. Based on these findings, several suggestions can be proposed. Internet recycling companies should leverage comparative advantages to meet PE and explore good profit models. Internet recycling companies should develop heterogeneous publicity strategies to expand the impact of SI, strengthen the construction of offline supporting facilities, and integrate informal recyclers to create FC for Internet recycling. Moreover, the government should increase financial support for recycling companies, and optimize the policy environment.

This study has some limitations. The exploration of the influencing factors in this study mostly focused on existing factors in the UTAUT model. However, with the development of Internet recycling, relevant influencing factors should be enriched based on the actual situation to analyze factors of Internet recycling participation in more detail. In addition, this study considered all types of Internet recycling platforms as a whole. However, existing Internet recycling models in China can be divided into three categories: recycler-led, disposer-led, and producer-led. These three types of Internet recycling platforms differ in their operation modes and recycling processes, and future research should design more targeted questionnaires to analyze the characteristics and differences of various types of Internet recycling platforms, allowing for more targeted suggestions for the development of Internet recycling.

## Figures and Tables

**Table 1:** The questionnaire structure with measurement indicators.

Constructs	Indicator Code	Indicators
Effort expectancy	EE1	I think it is easy to learn to use Internet platforms for recycling.
	EE2	I think Internet recycling is an easy thing to do.
	EE3	I know exactly how to recycle using the Internet platform.
Performance expectancy	PE1	Using the Internet recycling platform has saved time and improved efficiency for me.
	PE2	The Internet recycling platform provides me with timely and valuable recycling information.
	PE3	The Internet recycling platform has helped me a lot in my life.
	PE4	The Internet recycling platform provides me with personalized recycling services.
Social influence	SI1	I will try the Internet recycling platform if my family, friends, or colleagues recommend it.
	SI2	Mass media promotion would motivate me to try to use the Internet recycling platform.
	SI3	Support from related policies would lead me to use Internet recycling platform.
	SI4	Many people around me are using the Internet recycling platform.
Perceived risk	PR1	I am worried that Internet recycling will disclose my personal privacy information, location information, consumption information, etc.
	PR2	I am concerned about unreasonable charges or fraudulent spending when using Internet recycling methods.
	PR3	I am worried that learning Internet recycling will waste more of my time.
Facilitating conditions	FC1	I have the resources needed to use the Internet recycling platform.
	FC2	I have the knowledge needed to use the Internet recycling platform.
	FC3	Internet recycling is compatible with my previous recycling methods.
	FC4	If I have trouble using the Internet recycling platform, I can get help and guidance from someone (or a team).
Behavioral intentions	BI1	I am willing to keep learning about the use of new Internet recycling platforms.
	BI2	I would like to recommend Internet recycling to my family, friends, and colleagues.
Use behavior	UB1	I often use the Internet platform to recycle waste.
	UB2	I will continue to use the Internet recycling platform.
	UB3	I recommend Internet recycling to my family, friends, and colleagues.

**Table 2:** Sample distribution of the permanent resident population in Beijing.

Administrative Regions	Permanent Population (10,000)	Percent (%)	Sample Size
Total	2189.0	100%	500
1. Capital functional core area	181.5	8%	41
Dongcheng District	70.9	3%	16
Xicheng District	110.6	5%	25
2. Urban function expansion area	917.0	42%	209
Chaoyang District	345.1	16%	79
Fengtai District	201.9	9%	46
Shijingshan District	56.8	3%	13
Haidian District	313.2	14%	72
3. New urban development area	874.0	40%	200
Fangshan District	131.3	6%	30
Tongzhou District	184.0	8%	42
Shunyi District	132.4	6%	30
Changping District	226.9	10%	52
Daxing District	199.4	9%	46
4. Ecological conservation development area	216.5	10%	49
Mentougou District	39.3	2%	9
Huairou District	44.1	2%	10
Pinggu District	45.7	2%	10
Miyun County	52.8	2%	12
Yanqing County	34.6	2%	8

Data resource: Beijing Statistical Yearbook 2020, http://nj.tjj.beijing.gov.cn/nj/main/2021-tjnj/zk/indexch.htm (accessed on 11 February 2021).

**Table 3:** Main features of the respondents in Beijing (n = 500).

Characteristic	Group	Frequency	Percentage	S.E.
Gender	Male	240	48%	0.500
	Female	260	52%	
Age	<18	15	3%	0.469
	19–34	170	34%	
	35–49	145	29%	
	50–64	120	24%	
	>65	50	10%	
Education	Junior high school and below	10	2%	0.563
	High school	60	12%	
	Bachelor’s degree	380	76%	
	Master’s degree	45	9%	
	PhD	5	1%	

**Table 4:** Reliability and validity of the measurement model.

Constructs	Indicator Code	Loadings	Cronbach’s Alpha	C.R.	AVE
Effort expectancy	EE1	0.600	0.802	0.825	0.620
	EE2	0.955			
	EE3	0.767			
Performance expectancy	PE1	0.735	0.805	0.811	0.519
	PE2	0.759			
	PE3	0.719			
	PE4	0.664			
Social influence	SI1	0.848	0.803	0.817	0.529
	SI2	0.695			
	SI3	0.671			
	SI4	0.682			
Perceived risk	PR1	0.730	0.763	0.770	0.530
	PR2	0.812			
	PR3	0.630			
Facilitating conditions	FC1	0.753	0.804	0.807	0.511
	FC2	0.747			
	FC3	0.674			
	FC4	0.682			
Behavioral intentions	BI1	0.676	0.703	0.697	0.537
	BI2	0.785			
Use behavior	UB1	0.665	0.761	0.771	0.531
	UB2	0.795			
	UB3	0.720			

**Table 5:** Criteria and results of the fit index.

Fit Index	Reference Value	Model Value	Hypothesized Model Fit
CMIN/DF	<3 (Perfect) [45] <5 (Good) [46]	1.891	Yes
GFI	>0.9 [47]	0.916	Yes
IFI	>0.9 [48]	0.946	Yes
CFI	>0.95 (Perfect) >0.90 (Good) [49]	0.945	Yes
RMSEA	<0.05 (Perfect) <0.08 (Good) [50]	0.050	Yes
PNFI	>0.5 [51]	0.736	Yes

**Table 6:** Hypothesis testing.

Hypothesis	Path			S.E.	C.R.	*p*	Result
H1	BI	<---	EE	0.054	−0.392	0.695	Not supported
H2	BI	<---	PE	0.071	6.785	0.000 ***	Supported
H3	BI	<---	SI	0.047	3.19	0.01 **	Supported
H4	BI	<---	PR	0.025	−1.752	0.08	Not supported
H5	UB	<---	BI	0.056	3.504	0.000 ***	Supported
H6	UB	<---	FC	0.124	9.317	0.000 ***	Supported

Notes: *** indicates *p* < 0.001, ** indicates *p* < 0.01.

## Data Availability

The data investigated during this study are available from the corresponding author upon reasonable request.
